# Depression, Emotional Intelligence, and Lifestyle Correlates Among Medical Students in Pakistan: A Cross-Sectional Study Using the Beck Depression Inventory

**DOI:** 10.1192/bjo.2026.11208

**Published:** 2026-06-30

**Authors:** Minnahil Ali, Shahab Muhammad, Zainab Farooq

**Affiliations:** 1 HSE North Dublin Mental Health Services, Dublin, Ireland; 2 Pak Naval Services Shifa Hospital, Karachi, Pakistan; 3 HSE North Dublin City Mental Health Service, Dublin, Ireland

## Abstract

**Aims:**

This study explores the relationship between depressive symptoms and emotional regulation–specifically emotional attention, clarity, and repair–among Pakistani medical students, with an emphasis on gender-based differences.

**Methods:**

A cross-sectional study was conducted among 216 medical students at a single medical college in Pakistan. Participants completed the Beck Depression Inventory (BDI) and the Trait Meta-Mood Scale (TMMS). Data on demographics, physical activity, and hobbies were also collected. Gender-stratified chi-square analyses were used to assess associations between depression severity and emotional regulation.

**Results:**

Moderate to severe depressive symptoms were reported by 41.6% of students, while 58.4% exhibited minimal to mild symptoms. Females were significantly more likely to report insufficient emotional attention (12.6% vs. 2.5%,p=0.038), while males more frequently reported sports as a primary hobby and higher levels of vigorous physical activity. No significant gender differences were found in depression severity, emotional clarity, or emotional repair. Overall, 91.7% of students demonstrated adequate or excessive emotional attention, 82.4% had excessive clarity, but a striking 79.6% reported insufficient emotional repair. Lifestyle patterns were also concerning, with 58.3% of students engaging in less than two hours of moderate physical activity per week. Emotional repair was consistently poor across all depression categories and both genders, suggesting a universal deficit in emotion regulation rather than a gender-specific trend.

**Conclusion:**

High levels of depression and emotional dysregulation were observed across the student body, regardless of gender. Within Pakistan’s context of limited mental health services, academic pressure, and cultural stigma, these findings highlight a critical need for institutional support structures. Emotion regulation training, particularly targeting emotional repair, and broader access to psychological services should be prioritized. Given the sample was drawn from a single institution, the generalizability of findings is limited.

